# The Use of D-Optimal Mixture Design in Optimizing Formulation of a Nutraceutical Hard Candy

**DOI:** 10.1155/2023/7510452

**Published:** 2023-03-15

**Authors:** Zoubeida Souiy, Zahra Amri, Hussameddin Sharif, Aicha Souiy, Imed Cheraief, Khaled Hamden, Mouhamed Hammami

**Affiliations:** ^1^Biochemistry Laboratory, LR12ES05 “Nutrition-Functional Foods and Vascular Health”, Faculty of Medicine, University of Monastir, Tunisia; ^2^University of Zawia, Libya; ^3^Faculty of Medicine, University of Sfax, Tunisia; ^4^Laboratory of Bioresources: Integrative Biology and Valorization, Higher Institute of Biotechnology of Monastir, University of Monastir, Tunisia

## Abstract

The aim of this study was to optimize the formulation of hard candy with antiviral herbal extracts and flowered with *Citrus limon* peel essential oils. To substitute water fraction, the D-optimal mixture design was used. The optimized mixture fractions of the best hard candy formulation contain *Curcuma longa* extract (10%), *Artemisia herba-alba* Asso extract (3.33%), *Glycyrrhiza glabra* extract (1.66%), and *Zingiber officinale* extract (1.66%) and flowered by 20 *μ*L/100 gram of *Citrus limon* essential oils. The effect of the addition had been investigated on the sensory, physicochemical, and phytochemical of the hard candy according to the optimal formulation. The main component of *Citrus limon* essential oil is limonene (52.47%), which has a pleasant lemon fragrance. The mixture of herbal extract added increased the total phenols, the flavonoid, and the ash content of the formulated hard candy (10.90 ± 0.50 mg GAE/g, 0.054 ± 0.02 mg CE/g, and 0.018 ± 0.009, respectively). The measurement of the DPPH free radical activity reveals a good antioxidant activity (26.4%). Furthermore, the sensory analysis has shown a good appreciation. Thus, formulated hard candy is a sensorially and therapeutically interesting product.

## 1. Introduction

Since the emergence of the respiratory syndrome caused by SARS-CoV-2 infection, COVID-19 has spread to all the continents at an unprecedented pace. It is destroying not only the social life in the world, with a mortality rate that is increasing every day, but also the economy [1]. During this public health emergency, food nutraceuticals could be promising therapeutics for COVID-19. In these circumstances, immunostimulant and antiviral foods can provide protection against SARS-CoV-2, ensuring rapid recovery from COVID-19 and reducing the risk [2].

In fact, different plant antiviral compounds, including terpenoids, lignans, peptides, flavonoids, polysaccharides, polyacetylenes, and alkaloids, are effective against different targets of viruses, such as RNA genomes, DNA, membranes, ribosomal activity, and the replication process [3].

Traditional herbs are considered potential sources for treatment of COVID-19 [4]. Some of the early reports on the activity against COVID-19 included *Artemisia* spp. [5], *Curcuma longa* [6], *Glycyrrhiza glabra* [7], and *Zingiber officinale* [8].

*Artemisia* (family *Asteraceae*) is one of the most widely distributed genera. Species of *Artemisia* are widely consumed as tea, traditional food, and dietary supplements. In fact, they are rich in carbohydrates, fatty acids, protein, dietary fiber, essential amino acids, minerals, and vitamins [9]. The plants have different biological properties like antimalarial, antiparasitic, antiasthmatic, antiemetic, antihyperlipidemic, antiepileptic, antihypertensive, anxiolytic, antitubercular, antidiabetic, antidepressant, hepatoprotective, insecticidal, gastroprotective, anticancer, antiviral activities, and against COVID-19 [10].

Indeed, the genus *Artemisia* is known to contain many bioactive compounds such as artemisinin and artesunate. The WHO recommends artemisinin-based combination therapies (ACTs) as first-line treatments for uncomplicated *Plasmodium falciparum* malaria. For the treatment of COVID-19, Kapepula et al. reported that artesunate was associated with shorter duration of COVID-19 symptoms and hospital stays than the standard of care [5].

*Curcuma longa* is used as a coloring agent and a spice in food preparations [11]. Indeed, curcumin has been demonstrated to be safe in human trials [12]. It is known to have anti-inflammatory, antitumor, and antioxidant properties. The main active component is curcumin. This component and its new derivatives (Cucurcumin and gallium-curcumin) have an antiviral activity against diverse viruses, such as HSV-1, influenza, hepatitis, chikungunya (CHIKV) or Zika (ZIKV), herpes simplex 2 (HSV-2), human immunodeficiency (HIV), and human papillomavirus (HPV) [13]. Therefore, curcumin could be a potential treatment option for patients with COVID-19 [6]. In fact, the natural polyphenolic compound could modify various targets on molecular base which participate in attaching of COVID-19 [14]. It may also suppress fibrosis and pulmonary edema-associated pathways in the infection of COVID-19 [15]. Thus, it could be a potential prophylactic therapeutic for COVID-19 [16].

*Zingiber officinale* is commonly known as ginger (belonging to family *Zingiberaceae*) and is a very popular spice as well as a main ingredient in many traditional and folk medicines [17]. It reduces cold and cough and has anti-inflammatory, antinausea, and good digestive properties [18]. The active compound (*β*-sitosterol) contained in *Zingiber officinale* is predicted to have a potential antiviral activity, especially against COVID-19 [8].

Haridas et al. [8] indicated that the specific compounds present in *Citrus medica* and *Zingiber officinale* have significant affinity in silico to spike protein of virus and ACE-2 receptor in the host.

*Glycyrrhiza glabra* is used worldwide for the treatment of cough and cold [19]. The main active compounds are glycyrrhizin and glycyrrhetinic acid. It has an antiviral activity against coronavirus, respiratory syncytial virus, vaccinia virus, HIV-1, vesicular stomatitis virus, and arboviruses [7]. Phytoconstituents from *Glycyrrhiza glabra* were used as repurposed drugs against SARS-CoV-2 [20]. Glycyrrhizin was tested against SARS-CoV-2 isolates replicated in renal epithelial cells and was found to be the most convincing interceptor of SARS-CoV-2 replication compared to other antiviral agents such as mycophenolic acid, pyrazofurin, 6-azauridine, and ribavirin [21].

Essential oils (EOs) have long been known to have anti-inflammatory, antiviral, and immunomodulatory properties [22]. Indeed, they are being proposed to have activity against the SARS-CoV-2 virus. Owing to their lipophilic nature, EOs are advocated to penetrate viral membranes easily leading to membrane disruption. Moreover, EOs contain multiple active phytochemicals that can act synergistically on multiple stages of viral replication and induce positive effects on the host respiratory system including bronchodilation and mucus lysis [23].

Citrus essential oils have antibacterial, antielastase, anti-inflammatory, antibiofilm properties, and antiviral activity, particularly against coronavirus [24]. Thus, the potential of plants, plant extracts, and molecules derived from plants that have activity against SARS-CoV-2 could be used for a preventive or curative treatment against COVID-19.

Hard candy is one of the functional foods with shiny appearance and hard texture. It was prepared from various ratios of sucrose (43-85% *w*/*w*), glucose syrups (15-57% *w*/*w*), sweeteners, coloring agents, flavors, and organic acids. It was developed because of the sensory pleasure, simple manufacturing, and long shelf life.

In this article, we have consolidated the data of these medicinal plants (*Artemisia herba-alba* Asso, *Curcuma longa*, *Glycyrrhiza glabra*, *Zingiber officinale*, and *Citrus limon*) that show promising antiviral activities against coronavirus in other to prepare a hard candy enriched with a mixture of the herbal aqueous extracts and flowered by *Citrus limon* essential oils.

## 2. Materials and Methods

### 2.1. Plant Material

The aerial parts of *Artemisia herba-alba* Asso were collected from Jabal Sagoufta, Qafşah (at the southwestern of Tunisia), in April 2021. The plant material was dried in the open air, in the dark, and at room temperature for 2 weeks until constant weight. Dried *Curcuma longa*, *Glycyrrhiza glabra*, *Zingiber officinale*, and the peel of the lemon (*Citrus limon*) were obtained from a local market (Tunisia).

### 2.2. Preparation of Samples

#### 2.2.1. Preparation of the Aqueous Extract

In total, 10 grams of each powder was added to 100 mL of sterilized distilled water on a Bioblock Scientific Fisher Stuart Shaker for 24 h at room temperature. The extract was centrifuged (3000 rpm for 5 min) and filtered twice using a filter paper (Whatman No. 1).

The extract thus obtained was used as a crude extract and kept at 4°C for subsequent investigations.

#### 2.2.2. Extraction and Analysis of the Essential Oil

Dried samples (100 g and 800 mL of water, three times) were subjected to hydrodistillation for Clevenger in accordance with the European Pharmacopoeia method. To eliminate any trace of water in the essential oils (EOs), the recovered distillate is stored in the freezer (-20°C) for 24 hours. The water phase is frozen, and the essential oil is separated, determined, and stored at 4°C in dark glass bottles until used.

The analysis was carried out using GC 6890N and 5975B MS Agilent model, equipped with an Agilent Technologies capillary HP-5MS column (30 m × 0.25 mm i.d.) form of fused silica HP type 1 (0.25 *μ*m film thickness) and an electron collision ionization (70 eV ionization energy). The oil sample was directly injected. The gas chromatography was started at 50°C and maintained for 1 min at 260°C and then for 5 min with a flow rate of 7°C/min. The split mode was used with a ratio 1 : 100. The detector and injector temperatures were set at 280 and 250°C, respectively.

Identification of components was assigned by using the Wiley and NIST 16 library data standards.

#### 2.2.3. Hard Candy Preparation

The candy was made according to the protocol carried out by Akib et al. [25], modified. Sucrose was dissolved in water boiled at 100°C. Then, the glucose syrup was added. The mixture was baked at 110°C for 15 min. After cooling to a temperature of 50°C, the hard candy paste underwent kneading, shaping, and packaging [26]. The candy formulation was prepared according to the “Box–Behnken” design. Indeed, we have optimized the sensory quality by varying the three components of the hard candy (sugar, glucose syrup, and water). The best formulation was 50 g of sugar, 25 g of glucose syrup, and 15 mL of water. Then, the candy was flowered by adding a concentration of 20 *μ*L/100 g, *Citrus limon* essential oils. The addition of the essential oil was done during the cooling step, just before the candy crystallized.

### 2.3. Experimental Design

The aim was to formulate a new hard candy with herbal extracts to substitute water. The rates of each component (sugar, glucose syrup, and water) were chosen according to the formulation previously optimized. The rate of each extract was determined using the mixing plan, generated by the JMP statistical software package (version 14. Pro, SAS Institute, United Kingdom).

For this purpose, a two-level D-optimal mixing design with four factors was used to construct mathematical models for the sensory optimization process. The responses were the descriptors “texture, aroma, taste, color, and overall acceptability.” Table 1 shows the variables encoded for the mixture design approach of ten (*n* = 10) formulations. According to the D-optimal mixture design approach, the influence of these components on sensory quality was determined and an optimum combination was evaluated. For optimization, depending on the effect of each factor, the combination of factors that led to the best responses was evaluated.

### 2.4. Sensory Evaluation

The sensory optimization of the prepared formula was done by a hedonic test according to a detailed sheet on the criteria of the candies with a scale well defined by 20 panelists. Each test was replicated twice. The sensory rating scale ranged from (1) to (5) as follows: (1) do not like at all; (2) do not like; (3) neutral; (4) like; and (5) like a lot [27]. The result of statistical processing by the JMP 16 software (SAS, USA) gave us the mixture of extracts which resulted in sensory optimization.

### 2.5. Physicochemical Analyses of Hard Candy

The carried-out tests of samples consisted of pH, titratable acidity, moisture content, and ash content. Analysis was performed according to the standard instructions.

#### 2.5.1. pH Value and Titratable Acidity

The pH value of hard candies was evaluated by mixing 1 g of the hard candy with 100 mL of distilled water (50°C) and vortexed for 3 min. The suspension was held at room temperature for 1 h and centrifuged. The pH of the supernatant was determined by a digital pH meter. The titratable acidity was identified by titrating the suspension hard candy with NaOH (0.1 N) up to pH 8.1 [28].

#### 2.5.2. Moisture Content

The moisture content was identified according to the AOAC method [29]. The drying temperature was set at 105°C.

#### 2.5.3. Ash Content

The sample's ash content determines the mineral content. It was measured by the gravimetric method at a temperature of 525°C.

### 2.6. Phytochemical Content and Antioxidant Activity

#### 2.6.1. Total Phenol Content (TPC)

The method of Montedoro et al. [30] was used to measure the TPC. The TPC was determined from the calibration curve prepared by gallic acid. They were calculated as mg gallic acid equivalents (GAE)/g hard candy.

#### 2.6.2. Total Flavonoid Content (TFC)

The flavonoid content of the hard candy sample was measured using the aluminum chloride (AlCl_3_) method described by Ribarova et al. [31].

#### 2.6.3. Antioxidant Activity

The antioxidant capacity was identified using 1,1-diphenyl-2-picrylhydrazyl (DPPH), namely, free radical-scavenging activity, according to the method of Braca et al. [32].

### 2.7. Statistical Analysis

Each analysis of the candy sample was evaluated based on data determined from three replicates. The data were calculated as means ± standard deviation (SD), statistically evaluated by analysis of variance (ANOVA). The used test was Tukey's post hoc with a 5% risk level, using SPSS 26 software (IBM, United States).

## 3. Results and Discussion

The new formulation is aimed at replacing the percentage of water with a mixture of extracts and flowered by the *Citrus limon* peel essential oils.

### 3.1. Citrus Limon Peel Essential Oils

**Figure 1:**
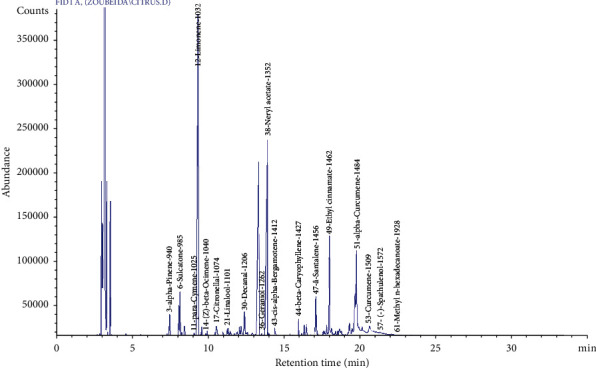
Chromatogram of *Citrus limon* peel essential oil components.

The essential oil obtained has a liquid and limpid appearance, having a light-yellow color with a fresh, fruity, and tangy odor. The yield of essential oil extraction from the *Citrus limon* peel is 0.65. This result is comparable to other studies [33] which found variable proportions of around 0.7%. On the other hand, other works like those of Guimarães et al. (2010) have shown that the Citrus peel essential oil yield is 0.18 ± 0.004% [34]; however, in Hamdan et al.'s work [35], the yield is 2%. This difference in yield is not only due to a set of parameters such as geographical origin, extraction method, time of extraction, and fruit maturity but also due to the conditions of experimentation [33]. Citrus peel essential oils have many medicinal properties and can be used in the treatment of various diseases [36]. The essential oil was extracted by hydrodistillation and analyzed by gas chromatography/mass spectroscopy (GC/MS). The chemical composition flowchart is shown in Figure 1. The chemical composition of the essential oils from *Citrus limon* peel is shown in Table 1.

In total, 61 volatile compounds, representing 98.68% of the oil, have been detected in *Citrus limon* peel essential oils. The EO is characterized by a high proportion of monoterpenes (57.43%), oxygenated monoterpenes (12.01%), terpene esters (11.16%), and sesquiterpenes (9.6%). However, we identify low percentages of ketone, aromatic compound, aliphatic aldehyde, oxygenated sesquiterpene, oxygenated sesquiterpene, and fatty acid esters representing 2.45%, 2.34%, 2.07%, 1.31%, 0.16%, and 0.15% of total HE, respectively.

The major components are limonene (52.47%), neryl acetate (10.6%), and nerol (8.63%). This is like most of the previous work which indicated that the essence of *Citrus limon* is composed of 92% to 93% of terpenes of which d-limonene is the most abundant [37]. Similarly, a total of forty-three compounds have been found by Paw et al. [38] including limonene (55.40%) and neral (10.39%) and were identified as major compounds followed by *trans*-verbenol (6.43%) and decanal (3.25%).

### 3.2. Analysis of D-Optimal Mixture Design

There are many types of mixture design of experiments, including simplex-centroid, simplex-lattice, D-optimal design, and axial design [39]. A D-optimal mixture design was employed to check out the influence of herbal extracts (*X*_1_: *Curcuma longa L* extract, *X*_2_: *Artemisia herba-alba* Asso extract, *X*_3_: *Glycyrrhiza glabra L* extract, and X4: *Zingiber officinale* extract) with the observed responses (color (Y1), taste (Y2), flavor (Y3), texture (Y4), aroma (Y5), and overall acceptability (Y6)). The main number of experiments (*n* = 10) was suggested by the JMP 16 software (SAS, USA), and the presentation of the samples was set at random (Table 2).

The goodness of fit (*R*^2^) and analysis of variance ANOVA were applied to test the fit of the linear models. This test involves that the sum of the squares of the residual response of the response was separated into the pure error and the model error. Their meanings were obtained by an *F*-test. The probability value (*p* value) for determining statistical significance was set at 0.05, indicating that a theoretical “hypothesis” would be rejected if the *p* value was less than 0.05.

**Figure 2:**
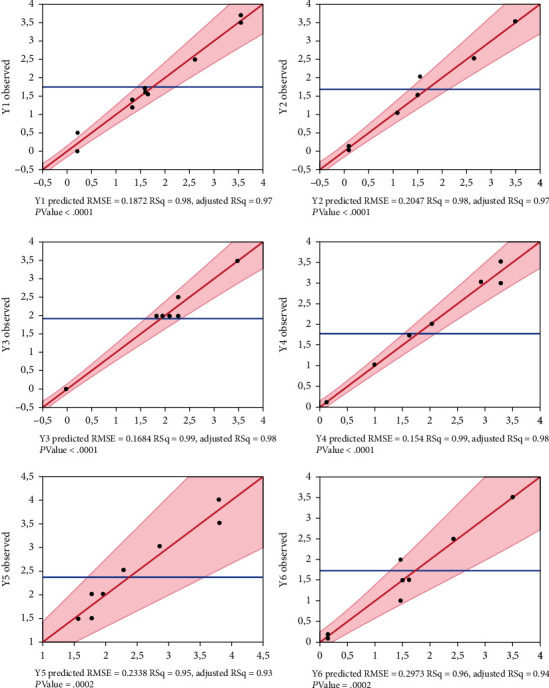
Graph of observed values versus predicted values. Y1: color, Y2: taste, Y3: flavor, Y4: texture, Y5: aroma; Y6: overall acceptability.

**Figure 3:**
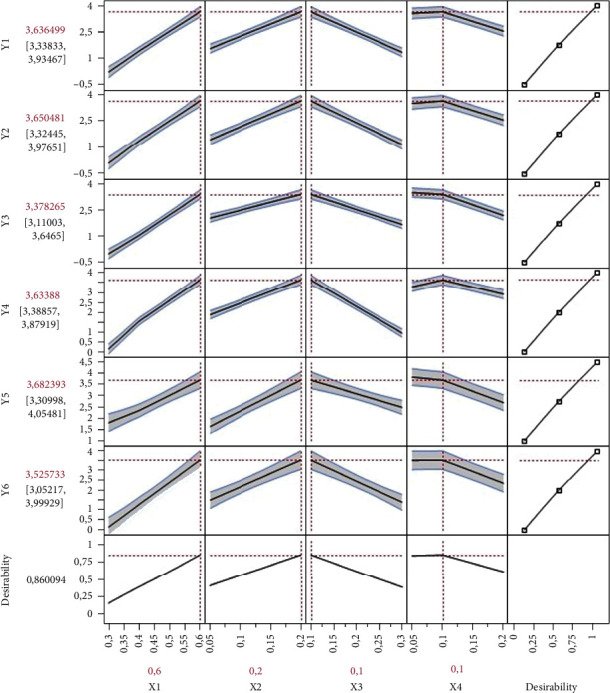
Maximum desirability in profiler for mixture analysis.

As seen in Figure 2, the values of adjusted *R*^2^ (0.97, 0.97, 0.98, 0.98, 0.93, and 0.94) and *R*^2^ (0.98, 0.98, 0.99, 0.99, 0.95, and 0.96) showed that the predicted values were in a good agreement with experimental data. The predictive mathematical model is defined in Table 3. The mathematical models were first-order regression models. Thus, the four factors have a significant effect on the formulation of the therapeutic candy; *X*_1_ (*Curcuma longa* L. extract) and *X*_2_ (*Artemisia herba-alba* Asso extract) were the most significant factors. The impact of the variables on organoleptic characteristics of the therapeutic candy formulations can be introduced by the effect diagram in Table 4. Table 4 shows that organoleptic characteristics improved mainly by an increased ratio of *Curcuma longa* L. extract (*X*_1_). A lower quantity of *Glycyrrhiza glabra* L. extract (mL) and *Zingiber officinale* extract (*X*_3_ and *X*_4_) is better for the organoleptic quality of the hard candy formulation. The best formulation could provide more nutrition with the total substitution of water by herb extracts.  Figure 3 describes the optimal conditions (9 mL for *Curcuma longa* L. extract, 3 mL for *Artemisia herba-alba* Asso extract (*X*_2_), 1.5 mL for *Glycyrrhiza glabra* L. extract (*X*_3_), and 1.5 mL for *Zingiber officinale* extract (*X*_4_)). Additional experiments with optimal conditions were performed to confirm the adequacy and validity of the predicted models.

The new preparation of the therapeutic candy is made using the previously optimized ingredients and is used for the preparation of the therapeutic candy and naturally flowered by *Citrus limon* peel essential oils. Our study shows that the substitution of water fraction in hard candy by a mixture of herbal extracts is possible.

### 3.3. Organoleptic Test

Hard candy hedonic test results identified that most panelists liked the candy-added anticoronavirus extracts. Then, from the ANOVA data, we can see that the hard candy and control samples (HC) were significantly different.

Table 5 shows the mean sensory ratings for the newly formulated hard candy (FHC) and control sample (HC) (without herbal aqueous extracts and not flowered by *Citrus limon* essential oils). There were significant differences in color, taste, flavor, texture, aroma, and overall acceptability between samples (*p* value was less than 0.05).

The mean score of judges for overall acceptability was significantly superior (*p* < 0.05) as compared to the control sample (3.5 to 2, respectively). The hard candy hedonic test results obtained most panelists liked the candy-added anticoronavirus extracts. The mean score of judges for color significantly decreased (*p* < 0.05) from 3.7 to 2.00. The hard candy (FHC) colors are yellow. The yellow color comes from the *Curcuma longa* L extract. The higher concentration added resulted in the colors of the hard candy.

Flavor determines the consumer's attraction to the food product. The judges detected the changes in the flavor profile of the newly formulated sample rated superior as compared to the control sample. The mean score of panelists for flavor significantly decreased (*p* < 0.05) from 3.5 to 1.70, respectively. In fact, the major component of the added essential oil was limonene, which is usually used as a flavor additive in food for its pleasant lemon-like odor.

### 3.4. Physicochemical Analyses

Results of the physicochemical analyses of the newly formulated hard candies (FHC) and the control sample (HC) are shown in Table 5.

#### 3.4.1. Determination of Moisture Content

As shown in Table 5, the moisture content of two hard candies (FHC and HC) was about 2. Based on these results, it has met specifications of ISO 3547.1: 2008 and is no more than 3.5% mass fraction. The moisture content in hard candy affects their appearance, structure, and texture. In fact, an increase in moisture content in hard candies within the range 1.3–2.0% was endorsed to decrease all the parameters investigated, such as cohesiveness, maximum cutting force, gumminess, elasticity, chewiness, and hardness [40]. However, an increase in moisture of over 2.0% did not cause a significant modification in candy texture [40]. However, high water levels cause humidity, which leads to easy contamination by fungi and bacteria [41]. The variation in moisture content can be explained by manufacturing conditions and storage time. The water content values influence the hardness of the candy. The higher water content of the candy caused the lower hardness value. The high water content will cause hard candy that is not hard enough and melts too easily [26].

#### 3.4.2. pH and Titratable Acidity

The taste of candies depends on their pH. At the same Brix degree of the first syrup, the pH of the newly formulated hard candy (FHC) and the control sample (HC) is 6.97 and 7.27, respectively. This result shows a nonsignificant effect of the herbal extracts on the pH of the candy. In fact, the acidity depends on the hydrolysis of sucrose to lactic acid, showing that increasing the temperature and supersaturation implicated a significant decrease in pH.

The combination of the mixture of the herbal extracts could have a synergistic reaction that probably leads to increased acidity of the candy.

The differences in our study and that of Kumar and Kirad [42] may be due to the formulation approach, candy preparation method, and nature of raw materials.

#### 3.4.3. Determination of Ash Content

Ashes are estimated the organic substance. Determination of ash content is one of the quality requirements on confectionary products. Usually, these components consist of calcium, potassium, iron sodium, magnesium, and manganese [25]. As shown in Table 5, the newly formulated hard candy (FHC) is richer in ash content than the control sample (HC). The mixture of herbal extract added increased the ash content value of hard candy. A high-purity glucose and sucrose syrup was used. The ash content is due to the mineral content in the *Curcuma longa* L. extract, *Artemisia herba-alba* Asso extract, *Glycyrrhiza glabra* L extract, and *Zingiber officinale* extract.

### 3.5. Phytochemical Content and Antioxidant Activity

#### 3.5.1. Total Phenol Content (TPC)

The antioxidant activity is mainly explained by the level of phenolic compounds [43]. The TPC obtained in the formulated hard candy (FHC) was higher than that obtained in the control sample (HC) (10.90 ± 0.50 mg GAE/g and 1.2 ± 0.50 mg GAE/g, respectively). This could be due to the richness of the aqueous extracts in phenolic composition. Therefore, we can confirm that the 15 mL of herbal extracts (60% *Curcuma longa* L. extract, 20% for *Artemisia herba-alba* Asso extract, 10% for *Glycyrrhiza glabra* L. extract, and 10% *Zingiber officinale* extract) had an influence on the total phenolic composition of the formulated hard candy. In fact, Tanvir et al. [44] found a total phenolic composition in *Curcuma longa* aqueous extracts ranging from 4.52% to 7.68%, while Mohamed et al. [45] demonstrated the richness of *Artemisia herba-alba* Asso extract phenolic composition (about 88 mg/g dry weight of phenolics).

#### 3.5.2. Total Flavonoid Content (TFC)

Flavonoids are the plant pigments that exert their health-promoting activities and are responsible for plant colors [44]. Referring to Table 5, we note that the total flavonoid contents (TFC), determined in the newly formulated hard candy (FHC), were significantly higher than those of the corresponding control sample (HC) (*p* < 0.05); the values are 0.054 ± 0.02 and 0.002 ± 0.001 mg CE/g dry weight, respectively. This could be explained by the richness of *Curcuma longa* extract in flavonoid composition. For example, Tanvir et al. [44] found a TFC of *Curcuma longa* varieties ranged between 0.29% and 0.67% in aqueous extracts. In addition, *Curcuma longa* from Malaysia has been reported to contain 0.094 mg/g of TFC of dry sample [46], while Sendi et al. [47] show that *Artemisia herba-alba* Asso is rich in health-promoting flavonoid compounds (80.035 mg CE/g dry matter).

#### 3.5.3. Antioxidant Activity

The concentrations of the newly formulated hard candy (FHC) and the control sample (HC) required to scavenge were 26.4% and 0.31%, respectively, of the DPPH free radical. This could be due to the richness of the cocktail of medicinal plants in a diversity of bioactive molecules. In fact, all the used herbal extracts showed very good antioxidant activity. Ramsewak et al. found that the antioxidant properties of curcumins 100 *μ*g/mL were 22 to 58% [48]. Labban [49] shows that extracts of *Curcuma longa* exhibit strong antioxidant activity, comparable to vitamins C and E.

In addition, concentration of a Tunisian *Artemisia herba-alba* extract is required to scavenge 50% of DPPH free radical [47]. The licorice aqueous extract showed strong antioxidant potential with IC_50_ (29.92 ± 2.43 mg/g) [50].

The obtained results indicate that the free radical-scavenging activity may be provided by the high contents of flavonoids and phenolics with a higher reducing capacity.

## 4. Conclusion

This research is focused on the production of health-promoting candies by using the nutraceutical potentials of *Curcuma longa*, *Artemisia herba-alba* Asso, *Glycyrrhiza glabra*, *Zingiber officinale*, and *Citrus limon* peel essential oil. This herbal extract could be a potential source of antiviral molecules against COVID-19; a D-optimal mixture design approach was used for the optimization of the formulation of new hard candies.

Results show that the candies produced have high contents of polyphenols, flavonoids, and ash. Measurement of anti-free radical activity reveals a good antioxidant activity.

This new product constitutes a promising preventive and therapeutic alternative to be assessed. So, the promotion of newly formulated candy enriched with traditional medicinal plants should be encouraged. However, further studies should be carried out to evaluate the clinical usefulness of this new product against COVID-19 infection. Furthermore, the mixture of the herbal extracts with possible anti-SARS-CoV-2 effects must be evaluated through prospective and interventional studies.

## Figures and Tables

**Table 1:** Chemical composition of the peel *Citrus limon* essential oil.

NP	Components	RT	*C* (%)	NP	Components	RT	*C* (%)
1	3-Hexen-1-ol	7.163	0.01	36	Geraniol	13.534	0.12
2	*alpha*-Thujene	7.311	0.13	37	Geranial	13.686	0.09
3	*alpha*-Pinene	7.471	1.2	38	Neryl acetate	13.922	10.6
4	*alpha*-Fenchene	7.739	0.02	39	1-Undecanol	13.976	0.15
5	*beta*-Pinene	8.077	1.62	40	*alpha*-Copaene	14.041	0.09
6	Sulcatone	8.170	2.45	41	Geranyl acetate	14.091	0.01
7	Myrcene	8.274	0.2	42	*beta*-Cubebene	14.162	0.03
8	*alpha*-Phellandrene	8.456	0.71	43	*cis*-*alpha*-Bergamotene	14.418	0.57
9	Delta-3-carene	8.769	0.01	44	*beta*-Caryophyllene	15.979	0.76
10	*alpha*-Terpinene	8.937	0.01	45	Carveolpropionate	16.346	0.55
11	*para*-Cymene	9.098	0.29	46	Zizaene	16.502	0.75
12	Limonene	9.365	52.47	47	*beta*-Santalene	17.130	2.29
13	1,8-Cineol	9.511	0.09	48	Seychellene	17.625	0.24
14	(Z)-*beta*-Ocimene	9.856	0.11	49	Ethylcinnamate	18.033	0.58
15	(E)-*beta*-Ocimene	9.954	0.32	50	Germacrene D	19.699	0.41
16	Gamma terpinene	10.421	0.06	51	*alpha*-Curcumene	19.809	0.72
17	*alpha*, p-Diméthylstyrène	10.559	0.36	52	*beta*-Bisabolene	20.322	0.59
18	Terpinolene	10.990	0.57	53	*beta*-Curcumene	20.637	1.93
19	*alpha*-Pineneoxide	11.180	0.05	54	*beta*-Sesquiphellandrene	20.834	0.53
20	p-Mentha-2,4(8)-diene	11.258	0.39	55	*beta*-Bisabolene	21.117	0.69
21	Linalool	11.338	0.44	56	Ledol	21.242	0.17
22	Nonanal	11.467	0.28	57	(-)-Spathulenol	21.346	0.87
23	p-Mentha-2,8-dien-1-ol	11.531	0.19	58	Methylarachidonate	21.593	0.04
24	*trans*-Verbenol	11.666	0.06	59	*beta*-Bisabolol	21.639	0.27
25	Camphor	11.734	0.16	60	Ethyl p-methoxycinnamate	21.761	0.32
26	Citronellal	11.890	0.17	61	Methyl n-hexadecanoate	22.482	0.11
27	Terpinen-4-ol	11.944	0.23	27	Monoterpenes		57.43
28	*alpha*-Terpineol	12.086	0.56	28	Oxygenated monoterpenes		12.01
29	Estragole	12.192	0.79	29	Terpene esters		11.16
30	Decanal	12.412	1.79	30	Sesquiterpenes		9.6
31	Geraniol	12.524	0.16	31	Ketone		2.45
32	*trans*-Carveol	12.599	0.28	32	Aromatic compound		2.34
33	Citronellol	12.942	0.38	33	Aliphatic aldehyde		2.07
34	Nerol	13.346	8.63	34	Oxygenated sesquiterpene		1.31
35	Neral	13.442	0.01	35	Total identified		98.68

RT: retention time; *C*: content; NP: peak no.

**Table 2:** Observed responses for the four-component D-optimal mixture design.

Simples	*X*_1_		*X*_2_		*X*_3_		*X*_4_		Y1	Y2	Y3	Y4	Y5	Y6
	(%)	(mL)	(%)	(mL)	(%)	(mL)	(%)	(mL)						
1	60	9	5	0.75	30	4.5	5	0.75	1.2	1	2	1	2	2
2	60	9	5	0.75	15	2.25	20	3	1.6	1.5	2	2	1.5	1.5
3	30	4.5	20	3	30	4.5	20	3	0.5	0	0	0.1	2	0.1
4	60	9	20	3	15	2.25	5	0.75	3.5	3.5	3.5	3.5	3.5	3.5
5	50	4.5	20	3	10	1.5	20	3	2.5	2.5	2	3	3	2.5
6	30	4.5	20	3	30	4.5	20	3	0	0.1	0	0.1	1.5	0.2
7	60	9	5	0.75	15	2.25	20	3	1.7	1.5	2	2	1.5	1.5
8	60	9	20	3	15	2.25	5	0.75	3.7	3.5	3.5	3	4	3.5
9	60	9	5	0.75	30	4.5	5	0.75	1.4	1	2.5	1	2	1
10	51	7.65	13.50	2.025	22	3.3	13.50	2.025	1.55	2	2	1.7	2.5	1.5

*X*_1_: *Curcuma longa* extract; *X*_2_: *Artémisia herba-alba* extract; *X*_3_: *Glycyrrhiza glabra* extract; *X*_4_:*Zingiber officinale* extract. Y1: color; Y2: taste; Y3: flavor; Y4: texture; Y5: aroma; Y6: overall acceptability.

**Table 3:** Predicted models of the responses (color (Y1), taste (Y2), flavor (Y3), texture (Y4), aroma (Y5), and overall acceptability (Y6)).

Predicted models
$\text{Y}1=3.729\left(\frac{X_{1}-0.3}{0.5}\right)+5.121\left(\frac{X_{2}-0.05}{0.5}\right)-2.27\left(\frac{X_{3}-0.1}{0.5}\right)-1.378\left(\frac{X_{4}-0.05}{0.5}\right)$
$\text{Y}2=3.646\left(\frac{X_{1}-0.3}{0.5}\right)+5.322\left(\frac{X_{2}-0.05}{0.5}\right)-2.275\left(\frac{X_{3}-0.1}{0.5}\right)-1.34\left(\frac{X_{4}-0.05}{0.5}\right)$
$\text{Y}3=3.729\left(\frac{X_{1}-0.3}{0.5}\right)+5.121\left(\frac{X_{2}-0.05}{0.5}\right)-2.271\left(\frac{X_{3}-0.1}{0.5}\right)-1.378\left(\frac{X_{4}-0.05}{0.5}\right)$
$\text{Y}4=3.79\left(\frac{X_{1}-0.3}{0.5}\right)+4.44\left(\frac{X_{2}-0.05}{0.5}\right)-3.227\left(\frac{X_{3}-0.1}{0.5}\right)+0.274\left(\frac{X_{4}-0.05}{0.5}\right)$
$\text{Y}5=3.048\left(\frac{X_{1}-0.3}{0.5}\right)+6.508\left(\frac{X_{2}-0.05}{0.5}\right)+0.362\left(\frac{X_{3}-0.1}{0.5}\right)-0.99\left(\frac{X_{4}-0.05}{0.5}\right)$
$\text{Y}6=3.738\left(\frac{X_{1}-0.3}{0.5}\right)+4.873\left(\frac{X_{2}-0.05}{0.5}\right)-1.949\left(\frac{X_{3}-0.1}{0.5}\right)-1.792\left(\frac{X_{4}-0.05}{0.5}\right)$

**Table 4:** The effect of different combinations studied.

Term	Estimation	Standard error	*t* ratio	*p* value > ∣*t*∣
(a)				
*X*_1_ − 0.3/0.5	3.730	0.155	24.03	<0.0001
*X*_2_ − 0.5/0.5	5.121	0.372	13.76	<0.0001
*X*_3_ − 0.1/0.5	−2.271	0.307	−7.40	0.0003
*X*_4_ − 0.5/0.5	−1.378	0.372	−3.70	0.0100
(b)				
*X*_1_ − 0.3/0.5	3.647	0.169	21.49	<0.0001
*X*_2_ − 0.5/0.5	5.322	0.407	13.08	<0.0001
*X*_3_ − 0.1/0.5	−2.752	0.335	−8.20	0.0002
*X*_4_ − 0.5/0.5	−1.344	0.407	−3.30	0.0164
(c)				
*X*_1_ − 0.3/0.5	4.408	0.140	31.57	<0.0001
*X*_2_ − 0.5/0.5	3.083	0.335	9.21	<0.0001
*X*_4_ − 0.5/0.5	−1.917	0.335	−5.73	0.0012
*X*_3_ − 0.1/0.5	−0.861	0.276	−3.12	0.0206
(d)				
*X*_1_ − 0.3/0.5	3.790	0.128	29.68	<0.0001
*X*_2_ − 0.5/0.5	4.440	0.306	14.50	<0.0001
*X*_3_ − 0.1/0.5	−3.227	0.252	−12.79	<0.0001
*X*_4_ − 0.5/0.5	0.274	0.306	0.90	0.405
(e)				
*X*_1_ − 0.3/0.5	3.048	0.194	15.72	<0.0001
*X*_2_ − 0.5/0.5	6.508	0.465	14.00	<0.0001
*X*_4_ − 0.5/0.5	−0.991	0.465	−2.13	0.077
*X*_3_ − 0.1/0.5	1.362	0.383	0.95	0.381
(f)				
*X*_1_ − 0.3/0.5	3.738	0.246	15.16	<0.0001
*X*_2_ − 0.5/0.5	4.874	0.591	8.25	<0.0002
*X*_3_ − 0.1/0.5	−1.950	0.487	−4.00	0.0071
*X*_4_ − 0.5/0.5	−1.793	0.591	−3.03	0.0230

**Table 5:** Organoleptic, physicochemical analyses, phytochemical content, and antioxidant activity.

Analyses	FHC	HC
	Mean value ± SD	
Color	3.7 ± 0.50	2 ± 0.40
Taste	3.5 ± 0.60	2.5 ± 0.60
Flavor	3.5 ± 0.40	1.7 ± 0.40
Texture	3 ± 0.55	1.6 ± 0.52
Aroma	4 ± 0.54	1 ± 0.50
Overall acceptability	3.5 ± 0.62	2 ± 0.64
PH	6.97 ± 0.20	7.86 ± 0.15
Titratable acidity (%)	0.032 ± 0.01	0.030 ± 0.009
Moisture content	1.99 ± 0.35	2.21 ± 0.40
Ash content (%)	0.018 ± 0.009	0.003 ± 0.01
Total phenol (TPC) mg GAE/g of dry weight	10.90 ± 0.50	1.2 ± 0.40
Total flavonoid content (TFC) mg CE/g dry weight	0.054 ± 0.02	0.002 ± 0.001
Antioxidant activity (%)	26.4 ± 0.40	0.31 ± 0.20

## Data Availability

Data will be available based on request to the corresponding author.

## Additional Points

*Practical Applications.* Nowadays, there is a strong demand for healthy food products. It would expand the options for developing food products that contain functional ingredients. Herbal extract is an attractive ingredient for these purposes, as it can be easily incorporated into various food formulas. This study is aimed at formulating a nutraceutical hard candy with herbal extract and flowered with *Citrus limon* peel essential oils. Water fraction was substituted by a mixture of four antiviral herbal extracts (*Curcuma longa* extract, *Artemisia herba-alba* Asso extract, *Glycyrrhiza glabra* extract, and *Zingiber officinale* extract). Results identified by most panelists liked the candy-added herbal extracts. The tested newly formulated hard candy was rich in total phenol content and total flavonoid content and exhibited a good antioxidant activity. The obtained new formulation showed a nonsignificant effect of the herbal extracts on the physicochemical analyses.
